# Direct Targeting Options for STAT3 and STAT5 in Cancer

**DOI:** 10.3390/cancers11121930

**Published:** 2019-12-03

**Authors:** Anna Orlova, Christina Wagner, Elvin D. de Araujo, Dávid Bajusz, Heidi A. Neubauer, Marco Herling, Patrick T. Gunning, György M. Keserű, Richard Moriggl

**Affiliations:** 1 Institute of Animal Breeding and Genetics, University of Veterinary Medicine, 1210 Vienna, Austria; anna.orlova@vetmeduni.ac.at (A.O.); Christina-Maria.Wagner@vetmeduni.ac.at (C.W.); Heidi.Neubauer@vetmeduni.ac.at (H.A.N.); 2 Department of Chemical and Physical Sciences, University of Toronto Mississauga, Mississauga, ON L5L 1C6, Canada; e.dearaujo@mail.utoronto.ca (E.D.d.A.); patrick.gunning@utoronto.ca (P.T.G.); 3 Centre for Medicinal Chemistry, University of Toronto Mississauga, Mississauga, ON L5L 1C6, Canada; 4 Medicinal Chemistry Research Group, Research Centre for Natural Sciences, H-1117 Budapest, Hungary; bajusz.david@ttk.mta.hu (D.B.); keseru.gyorgy@ttk.mta.hu (G.M.K.); 5 Department I of Internal Medicine, Center for Integrated Oncology (CIO), Excellence Cluster for Cellular Stress Response and Aging-Associated Diseases (CECAD), and Center for Molecular Medicine Cologne (CMMC), Cologne University, 50937 Cologne, Germany; marco.herling@uk-koeln.de

**Keywords:** STAT3, STAT5, cancer, small-molecule inhibitors

## Abstract

Signal transducer and activator of transcription (STAT)3 and STAT5 are important transcription factors that are able to mediate or even drive cancer progression through hyperactivation or gain-of-function mutations. Mutated STAT3 is mainly associated with large granular lymphocytic T-cell leukemia, whereas mutated STAT5B is associated with T-cell prolymphocytic leukemia, T-cell acute lymphoblastic leukemia and γδ T-cell-derived lymphomas. Hyperactive STAT3 and STAT5 are also implicated in various hematopoietic and solid malignancies, such as chronic and acute myeloid leukemia, melanoma or prostate cancer. Classical understanding of STAT functions is linked to their phosphorylated parallel dimer conformation, in which they induce gene transcription. However, the functions of STAT proteins are not limited to their phosphorylated dimerization form. In this review, we discuss the functions and the roles of unphosphorylated STAT3/5 in the context of chromatin remodeling, as well as the impact of STAT5 oligomerization on differential gene expression in hematopoietic neoplasms. The central involvement of STAT3/5 in cancer has made these molecules attractive targets for small-molecule drug development, but currently there are no direct STAT3/5 inhibitors of clinical grade available. We summarize the development of inhibitors against the SH2 domains of STAT3/5 and discuss their applicability as cancer therapeutics.

## 1. Introduction

The Janus kinase/Signal transducer and activator of transcription (JAK-STAT) pathway is one of the core cancer pathways that integrates signals from cytokines, hormones and growth factors to induce or repress gene expression in cells [1]. The pathway consists of four JAK kinases (JAK1, JAK2, JAK3 and TYK2) and seven STAT transcription factors (STAT1, STAT2, STAT3, STAT4, STAT5A, STAT5B and STAT6). Despite conserved structure and a common mechanism of action, STAT family members show distinct and even opposite functions in tumor biology. 

STAT1 is generally not associated with promoting tumor growth and mostly mediates tumor-suppressive and pro-apoptotic functions [2,3]. Consistently, STAT1^−/−^ mice are more prone to tumor development and *STAT1* deletion in leukemic cells decreases MHC class I expression [4,5]. Surprisingly, in a *v-abl*-driven model of STAT1^−/−^ leukemic cells initially harboring low MHC class I, enhanced MHC class I expression was gained during the disease progression, thereby reducing tumor recognition by NK cells [6]. As an exception, STAT1 was shown to be an oncogenic driver in T-cell acute lymphoblastic leukemia (T-ALL) and ALK^+^ anaplastic large cell lymphoma (ALCL), and STAT1 is associated with the JAK2 exon 12 mutation in the progression of myeloproliferative neoplasms (MPNs) [7,8,9]. Reports on direct involvements of STAT2, STAT4 and STAT6 in cancerous processes are scarce and are not discussed here further [10]. Associations of STAT3 and STAT5 with cancer progression are well established and heavily studied, and hence, are the main focus of this review.

## 2. Role of STAT3 and STAT5 in Cancer

STAT3 and STAT5 proteins are of particular interest in cancer research, as their hyperactivation was reported in processes ranging from inflammation and autoimmunity to infection and cancer. Within the latter, these proteins have been implicated in tumor initiation, as well as in metastasis or in conferring drug resistance mechanisms [10,11,12]. Constitutive hyperactivation of STAT3 and STAT5, as a result of their gain-of-function mutations or via enhanced signaling from upstream drivers, promotes tumor cell growth and survival [3]. 

STAT3 is the best-studied family member of the JAK-STAT pathway in cancer, and is a known oncogene in various types of solid malignancies, like melanoma or lung cancer [13,14,15]. Furthermore, both STAT3 and STAT5 are reported to play a major role in the progression and pathogenesis of prostate cancer [16,17,18]. Hyperactivation of STAT3 and STAT5 is commonly associated with an upstream oncogenic driver, such as hyperactive mutated tyrosine kinases, for example, JAK2^V617F^ or FLT3-ITD, or fusion proteins such as BCR-ABL, TEL-JAK2 or TEL-ABL1 [3,19,20,21]. The role of STAT5 in the transformation process induced by BCR-ABL p210 fusion protein has been particularly well-studied. It was shown that the absence of STAT5 diminishes the ability of cells to transform even upon harboring potent oncogenes such as BCR-ABL. Inducible deletion of STAT5 arrests and kills chronic myeloid leukemia (CML) cell lines, defining STAT5 as a therapeutic cancer target [22]. Hyperactivation of STAT3 and STAT5 can also occur via direct mutation in these genes, which is also associated with cancer progression in patients [1]. Interestingly, mutated STAT3 is mainly associated with large granular lymphocytic T-cell leukemia (T-LGLL), whereas mutated STAT5B is found in patients with T-cell prolymphocytic leukemia (T-PLL), T-ALL, γδ T-cell-derived lymphoma and monomorphic epitheliotropic intestinal T-cell lymphoma (MEITL) [23,24,25].

Inhibitors of kinases upstream of STAT3/5 are available, but patients often relapse by developing drug resistance through persistent signaling or enhanced upregulation of STAT3 or STAT5 expression [21,26]. Targeting STAT3 and STAT5 directly or in combination with tyrosine kinase inhibitors might be an attractive way to overcome these resistance mechanisms [27]. 

## 3. Non-Canonical Functions of STAT3 and STAT5 

**Figure 1:**
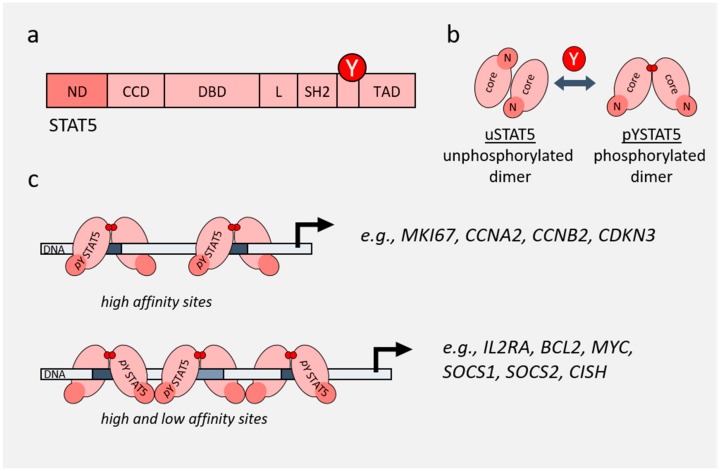
Functions of STAT5. (**a**) Domain structure of STAT5. Position of the critical activating tyrosine phosphorylation site is depicted with Y. ND—N-domain; CCD—coiled-coil domain; DBD—DNA-binding domain; L—linker; SH2—SH2 domain; TAD—transactivation domain. (**b**) Conformational changes of STAT5 from an unphosphorylated antiparallel dimer (uSTAT5) to a phosphorylated parallel dimer (pYSTAT5). (**c**) Dimer and oligomer conformations of STAT5 result in binding to GAS sites on DNA with different affinities, resulting in expression of different genes. Examples of dimer versus tetramer target genes are incorporated from [32,33].

STAT proteins share similar structural architecture including five domains: an N-terminal domain, a coiled-coil domain, a DNA-binding domain, an SH2 domain and a C-terminal transactivation domain [11] (Figure 1a). Upon phosphorylation, STAT5 undergoes a conformational change and forms parallel dimers via the SH2 domain (Figure 1b). This conformation allows the dimers to be recognized by importins and facilitates transport into the nucleus, where they bind GAS consensus sequences to induce target gene transcription. Such dramatic conformational rearrangements are possible due to the flexible linkers that are connecting the core fragment of STAT5 with its N- and C-terminus [28,29,30]. The complex approach of molecular dynamic simulations and bioinformatic analyses identified three distinct interaction surfaces within the dimer unique to STAT5, which include intramolecular interactions between the SH2 domain and the phosphotyrosine motif [28,31].

Interestingly, the SH2 domain of the hyperactivated gain-of-function variant STAT5B^N642H^ was also crystallized and compared to wild type human STAT5B. The crystal structure revealed two conformations of the mutant SH2 domain: one conformation is more closed, potentially allowing longer tyrosine phosphorylation lifetimes by blocking phosphatase attack. The other conformation, preserved in the crystal structure, revealed a more open conformation, which could facilitate phospho-peptide or protein interactions leading to hyperactivation [34].

Interestingly, the functions of STAT proteins are not limited to their state as phosphorylated dimers. Unphosphorylated STAT dimers (uSTAT), as well as tetramer/oligomer conformations, are involved in the functionality of some STATs [32,35]. Two phosphorylated dimers can form tetramers via their N-terminal oligomerization domains. This interaction stabilizes DNA binding and allows attraction of the tetramers to low-affinity sites, thereby providing altered binding selectivity and fine-tuning of transcriptional responses [36] (Figure 1c). 

STAT5 is particularly known for forming oligomers, a function shared with STAT1 and STAT4. In contrast, STAT3 shows weaker tetramer formation upon activation. STAT2 and STAT6 were not reported to form oligomers in cellulo [32,37,38,39]. However, yeast hybridization assays showed that recombinant N-domains of all STATs are able to self-dimerize [40]. Interestingly, this interaction is clearly homotypic, which might facilitate individual functions of different STATs. In addition, recombinant STAT2 and STAT6 N-domains can form oligomers with the same affinity as other STAT members, but this was not observed in cellulo [40]. 

The N-terminus also plays an important role in the function of STAT3: it mediates dimerization of uSTAT3, whereas for phosphorylated STAT3 (pYSTAT3) it can enable tetramerization [41,42]. Interestingly, oligomerization of STAT3 is not commonly observed in cellulo. Still, expression of certain genes like A2M (α2-macroglobulin) was shown to depend on an N-domain interaction of STAT3 [42]. 

Hu et al. showed that in STAT3^−/−^ MEFs, exogenous expression of an N-terminally truncated STAT3 protein leads to a decrease in expression of a small subset of genes, compared to expression of wild type STAT3 [43]. Not surprisingly, mutated STAT3^Y705F^, which is unable to become phosphorylated and to form parallel dimers, significantly affected global gene expression. However, certain genes (e.g., *MRAS*, *MET*) were still activated even by uSTAT3 [44]. Interestingly, STAT3^Y705F^ was able to weakly bind to selected promoters and to induce gene expression, thereby promoting an anti-viral, anti-proliferative effect in response to interferon stimulation. It would be interesting to determine if this activity is connected to the heterodimerization of STAT3 with STAT1 or if it is fully mediated through uSTAT3 [45]. uSTAT3 was also reported to be involved in interactions and complex formation with unphosphorylated nuclear factor kappa-light-chain-enhancer of activated B cells (NF-κB), which resulted in activation of a subset of NF-κB-dependent genes. Of note, the authors showed an additional subset of uSTAT3-dependent, but NF-κB-independent genes [46]. 

Timofeeva et al. showed that uSTAT3 plays a repressive role in the apoptosis of cancer cells and that inhibition of STAT3 N-domain functions can abolish this repressive effect. Treatment with the STAT3 N-domain peptidomimetic, ST3-H2A2, hindered STAT3 binding to the regulatory domains of various genes, including the gene for the proapoptotic C/EBP-homologous protein (CHOP). This, in turn, led to a decrease in the heterochromatin mark H3K9me3 in the promoter region of this gene [47]. 

uSTAT5 is mostly localized in the cytoplasm in the form of anti-parallel dimers. In mammalian cells, cytoplasmic uSTAT5 was shown to associate with and stabilize the Golgi apparatus [48]. On the other hand, a smaller proportion of uSTAT5 was also found in the nucleus where it colocalizes with the transcriptional repressor CTCF, thereby diminishing megakaryocyte differentiation via competition for DNA binding with the transcription factor ERG [35]. Additionally, there is evidence that uSTAT5 can migrate into the nucleus and bind heterochromatin protein 1α (HP1α) to promote the formation of heterochromatin. This leads to repression of various genes, among which many are found to be involved in cancer development [49]. 

One function of the STAT5 N-terminal domain involves docking of STAT5 to receptors. For example, it was shown that STAT5 interacts via the N-terminal domain with the glucocorticoid receptor [50]. The N-domain of STAT5 has an O-GlcNAc modification site on threonine 92. For STAT5A, it was shown that the presence of this glucose-derived modification within the N-domain is essential for full activation, suggesting cross-talk between the N- and C-domains and involvement of the N-domain in the regulation of metabolic functions in cells [51]. 

Another function of the STAT5 N-domain is mediating STAT5 tetramerization. Tetramerization was shown to be essential for proper T-cell development and was also found to be associated with enhanced activity of STAT5 as an oncogene [32]. STAT5 tetramers can bind to different motifs compared with STAT5 dimers, to induce or repress gene expression (Figure 1c) [52]. It was shown that deletion of the N-domain of STAT5 results in an absence of *c-MYC*, *BCL-2* and *cyclin D2* expression upon stimulation, indicating that oligomers can induce a different subset of genes than dimers [32]. Extensive studies by Lin et al., using a mutated STAT5 N-domain that is unable to form oligomers in vivo, defined the oligomer-dependent subset of genes as well as the importance of oligomerization in NK cell maturation [33,52]. 

The STAT5 N-domain was shown to be essential for leukemogenic transformation. Deletion of the N-domain or mutation of the O-GlcNAc-modified residue (T92A) abolished the initiation of the leukemic disease driven by gain-of-function STAT5A [32,51,53]. This suggests an important function of the STAT5 N-domain in oncogenic transformation and it proposes that the N-domain can serve as a novel targeting interface of STAT5.

## 4. Role of STAT3/5 in Chromatin Landscape

**Figure 2:**
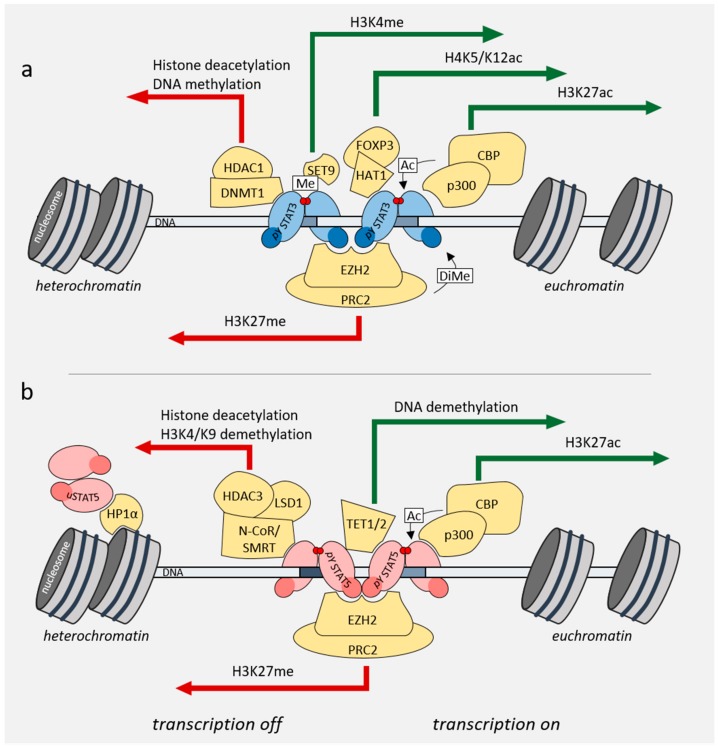
Role of STAT3/5 in regulating the chromatin landscape. (**a**) pYSTAT3 interacts with and recruits chromatin remodelers, and thereby promotes changes in chromatin compaction. (**b**) uSTAT5 and pYSTAT5 interact with different chromatin remodelers, thereby promoting chromatin changes associated with euchromatin (green arrows) or heterochromatin (red arrows) formation.

Over the last years, it was shown that STAT3 and STAT5 transcription factors can influence gene expression not only directly by binding to gene promoters but also through recruiting various chromatin remodelers and influencing gene expression and chromatin states on the global level. STAT3/5 can change the chromatin landscape in a cell by recruiting various chromatin-remodeling or DNA-modifying enzymes to the DNA, such as histone acetyltransferases (HATs), histone deacetylases (HDACs), as well as ten-eleven translocation methylcytosine dioxygenase 1/2 (TET1/2) or DNA (cytosine-5)-methyltransferase 1 (DNMT1) [54,55,56,57] (Figure 2a,b). These interactions influence eu- or hetero-chromatin formation or DNA methylation, thereby activating or repressing transcription. The STATs themselves can also be post-translationally modified by these enzymes: for example, methylation of STAT3 by the enhancer of zeste homolog 2/polycomb repressive complex 2 (EZH2/PRC2), or acetylation of STAT3 and STAT5 by CREB-binding protein (CBP)/p300 [1].

In the case of STAT3, acetylation or methylation at different residues leads to different activity and functionality of the protein (Figure 2a) [12]. For example, acetylation of STAT3 by CBP/p300 on lysine 685 in the C-terminal domain increases the DNA binding ability of STAT3 [58]. On the other hand, methylation of phosphorylated, promoter-bound STAT3 on lysine 140 by SET9 reduces its transcriptional activity on a subset of target genes [59], whereas dimethylation on lysine 49 by EZH2 is required for the expression of IL-6-dependent genes [60]. It was also shown that STAT3 binds to the promoter of the tyrosine phosphatase *SHP-1* and recruits DNMT1 and HDAC1 to silence its transcription in cutaneous T-cell lymphoma (CTCL) and in ALK^+^ ALCL cell lines [57]. In regulatory T-cells, STAT3 was found to cooperate with FoxP3 and HAT1 to induce expression of IL-10 [61]. 

STAT5 is also known to recruit chromatin remodelers (Figure 2b). One example of such an interaction is co-activation of STAT5 by HAT nuclear receptor coactivator 1 (NCoA-1). NCoA-1 and STAT5A transiently co-transfected in HEK293T cells were shown to interact with each other by co-immunoprecipitation. This interaction required amino acids 751 to 753 in the STAT5 transactivation domain, which is conserved in both STAT5A and STAT5B [54]. Furthermore, in Ba/F3 cells, STAT5A was shown to interact with HDAC3 and lysine-specific demethylase 1 (LSD1), thereby activating or repressing gene expression [55]. The interaction between STAT5A and LSD1/HDAC3 is mediated by the STAT5A DNA-binding domain, linker and its SH2 domain and HDAC3 can additionally interact with the coiled-coil domain of STAT5A [55]. STAT5 influences not only the acetylation but also the methylation status of its surroundings, for example by recruiting TET1/2 to the *Foxp3* locus. This causes demethylation of the locus and plays an important role in regulatory T-cell differentiation [56]. 

Mandal et al. showed that during B-cell maturation and B-cell receptor rearrangement, gene regions required for immunoglobulin κ light chain expression are silenced by histone methyltransferase EZH2-STAT5 tetramer interactions (Figure 2b) [62]. This underlines the importance of the STAT5 N-terminal domain and tetramerization during B-cell development or as a mechanism for acute B-cell leukemia initiation or progression. 

## 5. Direct STAT Targeting of the SH2 Domain

Treating cancers with hyperactivated or mutated STAT3 and STAT5 is currently achieved by targeting upstream kinases. However, despite tyrosine kinase inhibitors being significantly superior to classical chemotherapy, their application often causes severe side-effects and development of resistance [63]. Therefore, development of more specific and effective inhibitors that also target downstream components of hyperactivated pathways is desirable to overcome limitations of current strategies. In the following section, we focus specifically on small-molecule inhibitors of the STAT3/5 SH2 domains.

### 5.1. STAT3 Inhibitors

**Figure 3:**
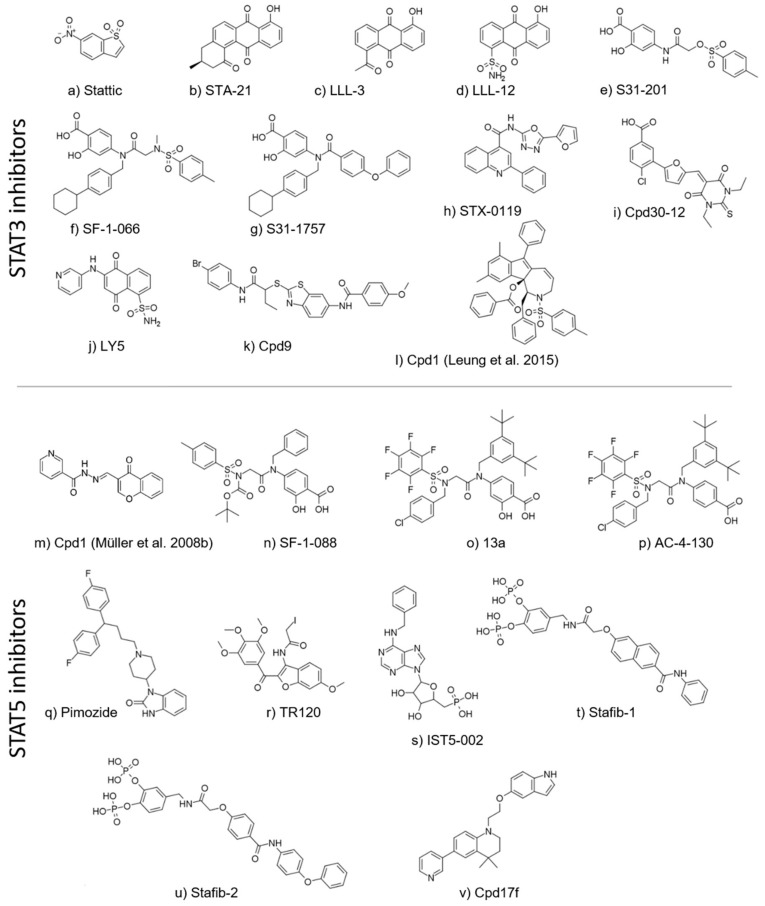
Chemical structures of STAT3 and STAT5 inhibitors (in their order of citation in the main text). Compounds with identical names (Cpd1) are further specified by citations.

The first two small-molecule STAT3 inhibitors were fragment-sized compounds discovered by random biochemical and virtual screening. One compound, Stattic, was identified in a high-throughput screen of a diverse chemical library (Figure 3a) [64]. Another anthraquinone-based compound, STA-21 (Figure 3b), was discovered by structure-based virtual screening against the published X-ray structure of STAT3β [65,66]. Later, multiple analogs of STA-21 were reported, such as LLL-3 and LLL-12 (Figure 3c,d) [67,68]. Recently, several different chemotypes have emerged, often consisting of ring systems connected by amide-containing linkers. An important step forward was the identification of the salicylic acid moiety that is an efficient bioisostere of the phosphate group required for STAT-STAT dimer formation. Salicylic acid analogs were described as potent STAT3 inhibitors as exemplified by the inhibitor S31-201 (Figure 3e), as well as its optimized successors, SF-1-066, SF-1-121 and S31-1757 (Figure 3f,g) [69,70,71]. 

Virtual screening was useful to find new chemotypes of STAT3 inhibitors. Matsuno et al. and Xu et al. described further double-digit micromolar STAT3 inhibitors, STX-0119 and Cpd30-12 (in cellular assays; Figure 3h,i) [72,73]. Furthermore, a number of natural products (or analogs thereof) and antioxidants have been proposed and identified as potential inhibitors of STATs, most particularly STAT3, displaying even single-digit micromolar inhibitory activities [74,75,76,77,78].

Another noteworthy study reported an in silico fragment-based drug discovery approach, resulting in a single-digit micromolar STAT3 inhibitor, LY5 (in both cell-free and cell-based assays; Figure 3j), targeted towards the SH2 domain [79]. Recently, Zhang et al. identified benzothiazole as a novel scaffold among STAT3 inhibitors, resulting from a virtual screening of more than 200,000 compounds by a multistep protocol. In this study, four compounds were confirmed experimentally, with the benzothiazole-based compound 9 (Figure 3k) displaying a single-digit micromolar IC_50_ value against the IL-6/STAT3 signaling pathway [80]. Two further analogs were discovered by a similarity-based hit expansion. When the number of known STAT3 inhibitors reached a sufficient level to compile a training set for a three-dimensional (3D) pharmacophore-based virtual screening study, Leung et al. screened a small in-house dataset and tested five compounds, out of which one (Cpd1, Figure 3l) was experimentally confirmed in multiple STAT3-related endpoints [81].

The compounds OPB-31121, OPB-51602 and OPB-111077, which are substances from Otsuka Pharmaceuticals, were designed to inhibit STAT3 phosphorylation in cancer cell lines and xenograft models by targeting the STAT3 SH2 domain. Available studies with OPB-51602 indicate induction of STAT3 aggregation in autophagosomes. Additionally, there has been controversy on the activity of OPB-51602 against mitochondrial STAT3. Two of these STAT3 SH2 inhibitors, OPB-31121 and OPB-51602, were already tested in phase I/II clinical trials for solid tumors, non-Hodgkin lymphoma, myeloma and other hematopoietic malignancies [81]. In these studies, the compounds were promising based on a relatively long half-life, suggesting the possibility of a reduced dosing regimen to limit toxic side effects. However, the clinical trials for both lead structure drugs were terminated due to minimal antitumor activity, toxicity issues and poor pharmacokinetics. Another STAT3 inhibitor, OPB-111077, has completed a phase I trial for solid cancers (NCT02250170), and phase I/II trials are recruiting for acute myeloid leukemia (AML) (NCT03197714) and other refractory tumors (NCT03158324) [82,83]. Efficacies of the above-mentioned STAT3 SH2 inhibitors in vitro and in vivo are summarized in Table 1. 

### 5.2. STAT5 Inhibitors

The first landmark papers on STAT5 SH2 domain inhibitors were published in 2008 by the Berg group. These were driven by the development of a robust high-throughput screening assay for STAT5B inhibitors [90,91]. This facilitated the discovery of chromone-based STAT5B inhibitors as well as a nicotinoyl hydrazine derivative, Cpd1 (Figure 3m), as a selective STAT5B inhibitor (in comparison to STAT1 and STAT3) [91]. 

Later, four salicylic acid-based compounds (including the single-digit micromolar inhibitor SF-1-088, Figure 3n) were identified by the Gunning group [92]. These hits were optimized to generate the STAT5B inhibitor, 13a, through a structure-guided approach (Figure 3o). Studies with 13a have shown it can effectively reduce pYSTAT5B levels in cellulo, and follow up thermal stability studies showed an inhibitor-induced reduction in STAT5 stability and the potential to block de novo phosphorylation [28,93,94]. Additional studies extended this work to demonstrate AML cell viability inhibition with AC-4-130, a further improved lead compound (Figure 3p) [95].

It is worth noting that in a 2011 study, Nelson et al. identified the neuroleptic drug pimozide as a STAT5 inhibitor (Figure 3q). However, recent studies have linked pimozide with proteolysis upstream of STAT5, rather than direct binding [96]. Nonetheless, the discovery later prompted Rondanin et al. to synthesize and screen a series of pimozide derivatives, two of which have surpassed the cytotoxic potency of pimozide as evaluated against imatinib-resistant BCR-ABL-expressing leukemia cells [97]. In 2016, the same group synthesized and tested 22 iodoacetamide-containing heterocycles for STAT5 inhibition, many of which (including TR120, Figure 3r) were confirmed experimentally in an in vitro cytotoxicity assay, although direct binding to STAT5 was not examined [98]. 

In 2015, Liao et al. conducted a large scale structure-based virtual screening campaign for STAT5A/B inhibitors [99]. Using a STAT3 based homology model of STAT5, the authors docked ~30 million compounds to the dimerization interface on the SH2 domain [65]. The top 30 hits were evaluated in various cell lines, with IST5-002 (Figure 3s) identified as a lead compound that inhibited STAT5A and STAT5B in the low micromolar range in cellulo. However, the first STAT5A/B gene product-selective inhibitor was developed in the same year, by the Berg group. Stafib-1 (Figure 3t), a catechol type bisphosphate-containing nanomolar STAT5B inhibitor (as evaluated in a fluorescence polarization assay), was identified by structure-based virtual screening against the SH2 domain of a STAT5B model derived from the structure of unphosphorylated STAT5A [30,100]. This compound was recently optimized into the single-digit nanomolar inhibitor Stafib-2 (Figure 3u) using a structure-based approach [101]. Stafib-2, similar to its predecessor, has shown high selectivity for STAT5B compared to other STAT proteins, including STAT5A. The hydrophilicity of the phosphate motifs was reduced by generating a pro-drug precursor, which employs pivaloyloxymethyl esters to conceal the negatively charged phosphate groups and increase cell penetrance. Recently, Natarajan et al. reported Stafia-1, a selective inhibitor for STAT5A, discovered by docking-based screening [102]. Tested applications for STAT5 inhibitors in vivo and in vitro are summarized in Table 2. 

Berg et al. also identified nucleotide scaffolds with potentially selective inhibition properties against STAT5B. Although, the IC_50_ values in a fluorescence polarization assay are in the high micro-molar range (STAT5B, IC_50_[ATP] = 97.4 ± 0.9 µM, IC_50_[GTP] = 95.5 ± 3.2 µM; STAT5A, IC_50_[ATP] = 443 ± 34 µM, IC_50_[GTP] = 311 ± 46 µM), these concentrations are still well below intracellular ATP concentrations (>2 mM). It would be of high interest to validate these findings in cellulo. These results suggest STAT5B may also play a role in directly coupling gene expression to cellular metabolism, and also highlight a potential new targeting strategy using nucleotide-based inhibitor scaffolds [104]. Recently, a novel mechanism of selectively blocking STAT5B activity was proposed through protein-mediated Mannich reactions. The ligand-efficient 4-amino-furazan-3-carboxylic acid molecule (K_d_ = 420 µM, STAT5B fluorescent polarization assay) was identified from a library of 17,000 compounds. This phosphate-mimetic reacts with 1*H*-tetrazoles in the presence of formaldehyde, and this reaction only proceeds in acidic conditions (pH 5.0). However, the addition of MBP-tagged STAT5B-SH2 domain peptide catalyzed the reaction even at physiological conditions. Although tetrazoles were not active against STAT5B (>10 mM), the ligation products showed substantial activity, which the authors attribute to super-additive binding interactions. Intracellular physiological concentrations of formaldehyde also emphasize the viability of this molecule as a STAT inhibitor and the utility of bio-catalytic Mannich reactions [105].

A study by Juen et al. reported the optimization of the STAT5 inhibitor, Cpd17f (Figure 3v) [103]. Interestingly, the initial hit was originally intended for PPARα/γ inhibition, while the inhibition of STAT5 phosphorylation was an off-target effect [106]. Since the compound did not inhibit PPARs, but showed a considerable inhibition of STAT5 phosphorylation, 18 analogs were synthesized and tested in a cell-based assay. As shown by Brachet-Bottineau et al., in this special issue, Cpd17f inhibits STAT5B protein expression through non-transcriptional mechanisms. However, as with the majority of the aforementioned inhibitors, further confirmatory assays demonstrating the mechanism of action are required. Although multiple studies using molecular modeling or computational dynamics have been insightful in STAT5 inhibitor design, protein-inhibitor complexes characterized with atomic-level resolution, such as through X-ray crystallization, would provide a clearer understanding of target engagement in efficacy and selectivity studies.

Apart from these efforts targeting the SH2 domain of STAT3 or STAT5 molecules, studies have also reported describing approaches to block STAT3/5 DNA binding or the use of antisense RNA interference, discussed further in this special issue with separate overview articles. 

## 6. Conclusions

STAT3 and STAT5 are the key nodes in transcriptional activation downstream of cytokine or kinase action in multiple cancers. This makes them attractive, but challenging targets for drug development. Recent findings expand on a previously rather simplistic understanding of STAT3/5 function as parallel phosphorylated dimers. It has become evident that higher-order conformations of pYSTATs, as well as uSTAT, are involved in chromatin landscape shaping, thereby acting beyond classical transcription factors. This knowledge provides a deeper understanding of their role in cancer biology, which together with various targeting efforts, will open novel therapeutic options.

## Figures and Tables

**Table 1:** Small-molecule compounds targeting the STAT3 SH2 domain.

Cpd	Protein-Based		Cell-Based			In Vivo Application Tested	Refs.
	Assay	IC_50_ or Ki [µM]	Cell Line	Readout	IC_50_ [µM]a		
Stattic	pY binding	5.1	HepG2, MDA-MB-231	viability	3.8		[64,84,85]
			RAW264.7	pYSTAT3	s (20)	Osteoclasto genesis in C57/BL6 mice (10 mg/kg)	
			M-SCC-17B, OSC-19, Cal33, UM-SCC-22	viability	2.2–3.5	Head and neck cancer xenograft (50 mg/kg)	
STA-21			Caov-3	reporter assay	s (20)	Psoriatic disease in mouse model and phase I in clinical trial (NCT0104794)	[66,86]
			MDA-MB-435s, MDA-MB-468, MDA-MB-231	DNA binding, viability	s (20)		
LLL-3			U373, MDA-MB-231	DNA binding	s (20)	Xenograft glioblastoma (50 mg/kg)	[68]
			MDA-MB-231	reporter assay	s (20)		
LLL-12			MDA-MB-231, SK-BR-3, PANC-1, HPAC, U87, U373, A549	viability, pYSTAT3, reporter assay	0.16–3.09	Glioblastoma, breast cancer xenograft (2.5, 5 mg/kg) Lung cancer xenograft (20, 10 mg/kg)	[67,87]
S31-201		80	NIH 3T3/v-Src	DNA binding	86	Xenograft breast cancer (5 mg/kg)	[69,71]
			MDA-MB-468, MDA-MB-231	pYSTAT3	s (100)		
			DU145, MDA468, OCI-AML-2	viability	28–112		
SF-1-066	FP	20	NIH 3T3/v-Src	DNA binding	35		[69]
			DU145, MDA468, OCI-AML-2	viability	17–37		
S31-1757	pY binding	13.5	HEK293	CoIP	s (50)		[88]
			MDA-MB-468, A549	pYSTAT3, reporter assay	s (50)		
STX-0119			HeLa	reporter assay	74	Xenograft lymphoma (160 mg/kg)	[73]
			HEK293	FRET-based dimerization	s (50)		
Cpd30-12	pY binding	114	HepG2, MEF/GFP-Stat3α, MDA-MB-468, MDA-MB-231, MBA-MD-435, MCF7	pYSTAT3, nuclear translocation, apoptosis	60		[72]
LY5	FP	2.5	U2OS, RH30, RD2, MDA-MB-231	viability, pYSTAT3	0.52–1.39	Xenograft breast cancer (5 mg/kg)	[79,89]
			UW426, UW288-1, DAOY	pYSTAT3	s (0.5)		
Cpd9			HepG2/STAT3	reporter assay, pYSTAT3	3.57		[80]
			MDA-MB-468	viability	8.83		
Cpd1	FP	~10	HeLa	reporter assay, DNA binding	~10		[81]

^a^ s (20): significant effect at 20 µM (or other concentration, as indicated); cpd—compound; FP—fluorescence polarization.

**Table 2:** Small-molecule compounds targeting the STAT5 SH2 domain.

Cpd	Protein-Based		Cell-Based			In Vivo Application Tested	Refs.
	Assay	IC_50_ or K_i_ [µM]^a^	Cell Line	Readout	IC_50_ [µM]^a^		
Cpd1	FP	47	K562, Daudi	DNA binding, pYSTAT5	s (100)		[91]
SF-1-088	FP	8.3	K562,MV4-11	viability, pYSTAT5	80–77		[92]
13a			K562	pYSTAT5, viability	s (15)		[28,93,94]
			MV4-11	viability	3.5		
AC-4-130	binding (thermal shift)	s (100)	MV4-11, MOLM-13	viability, reporter assay	1.7–1.9	AML xenograft (25 mg/kg)	[95]
			AML patient samples	viability	1.6–4.9		
Pimozide			KU812, K562	pYSTAT5, viability	s (5)	Approved by FDA as antipsychotic drug	[96]
TR120			K562	viability	0.12		[98]
				apoptosis	0.45		
IST5-002			K562, DU145, PC-3, COS-7	pYSTAT5, reporter assay, DNA binding	s (5)	Prostate cancer xenograft (25, 50, 100 mg/kg)	[99]
				viability	3.5		
Stafib-1	FP	0.044	K562	pYSTAT5	s (3)		[100]
Stafib-2	FP	0.009	K562	pYSTAT5	1.5		[101]
Cpd17f			K562, KU812, KG1a, MV4-11	viability, pYSTAT5	2.6–22.7		[103]

^a^ s (100): significant effect at 100 µM (or other concentration, as indicated); cpd—compound; FP—fluorescence polarization.
